# Structural basis for improved efficacy of therapeutic antibodies on defucosylation of their Fc glycans

**DOI:** 10.1111/j.1365-2443.2011.01552.x

**Published:** 2011-11

**Authors:** Tsunehiro Mizushima, Hirokazu Yagi, Emi Takemoto, Mami Shibata-Koyama, Yuya Isoda, Shigeru Iida, Kazuhiro Masuda, Mitsuo Satoh, Koichi Kato

**Affiliations:** 1 Graduate School of Pharmaceutical Sciences, Nagoya City University3-1 Tanabe-dori, Mizuho-ku, Nagoya 467-8603, Japan; 2 Antibody Research Laboratories, Kyowa Hakko Kirin Co., Ltd3-6-6 Asahi-machi, Machida-shi, Tokyo 194-8533, Japan; 3 Institute for Molecular Science and Okazaki Institute for Integrative Bioscience, National Institutes of Natural Sciences5-1 Higashiyama Myodaiji, Okazaki 444-8787, Japan; 4 GLYENCE Co., Ltd.2-22-8 Chikusa, Chikusa-ku, Nagoya 464-0858, Japan; 5 The Glycoscience Institute, Ochanomizu University2-1-1 Ohtsuka, Bunkyo-ku, Tokyo 112-8610, Japan

## Abstract

Removal of the fucose residue from the *N*-glycans of the Fc portion of immunoglobulin G (IgG) results in a dramatic enhancement of antibody-dependent cellular cytotoxicity (ADCC) through improved affinity for Fcγ receptor IIIa (FcγRIIIa). Here, we present the 2.2-Å structure of the complex formed between nonfucosylated IgG1-Fc and a soluble form of FcγRIIIa (sFcγRIIIa) with two *N*-glycosylation sites. The crystal structure shows that one of the two *N*-glycans of sFcγRIIIa mediates the interaction with nonfucosylated Fc, thereby stabilizing the complex. However, fucosylation of the Fc *N*-glycans inhibits this interaction, because of steric hindrance, and furthermore, negatively affects the dynamics of the receptor binding site. Our results offer a structural basis for improvement in ADCC of therapeutic antibodies by defucosylation.

## Introduction

The therapeutic applications of antibodies pioneered by von Behring & Kitasato (1890) at the end of the 19th century were revolutionized by the advent of monoclonal antibody technology, and subsequently, improved by genetic engineering approaches such as humanization (Mayforth 1993). To date, over 20 recombinant monoclonal antibodies have been licensed as drugs against various cancers and chronic diseases. Furthermore, antibody-based therapeutics currently account for most recombinant proteins in clinical use, and over 130 human monoclonal antibodies entered clinical trials between 2001 and 2008 (Pavlou & Belsey 2005; Reichert *et al.* 2005). Although the demand for industrial production of recombinant monoclonal antibodies is increasing, and the number of approved antibodies for therapeutic uses will increase during the next few years, one drawback of antibody medicine is the high cost of production. Production costs impose an economic limit on the therapeutic benefit of these approaches. Recently, a key breakthrough in the development of therapeutic antibodies has been achieved that may dramatically enhance effector functions through a cellular engineering technique for modifying the sugar chains displayed on the Fc region of immunoglobulin G (IgG) (Umaña *et al.* 1999; Kaneko *et al.* 2006; Jefferis 2009; Kubota *et al.* 2009; Yamane-Ohnuki & Satoh 2009).

**Figure 1:**
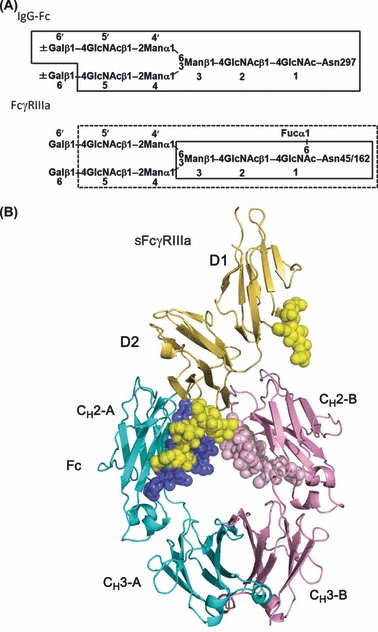
Structure of nonfucosylated Fc complexed with bis-glycosylated soluble form of Fcγ receptor IIIa (sFcγRIIIa). (A) *N*-glycans expressed on immunoglobulin G (IgG)-Fc and sFcγRIIIa used for crystallization. Sugar residues that gave interpretable electron densities are surrounded by solid polygons in Fc-Asn-297 *N*-glycans and sFcγRIIIa-Asn-162 *N*-glycan, whereas those in the sFcγRIIIa-Asn-162 *N*-glycan are indicated by a dashed box. (B) Overall view of the complex. Chains A and B of Fc are cyan and pink, respectively, and sFcγRIIIa is yellow. Carbohydrates are shown in sphere representation. F, Fuc, fucose; G, Gal, galactose; GN, GlcNAc, *N*-acetylglucosamine; M, Man, mannose.

The Fc region of IgG possesses a conserved glycosylation site at Asn-297 in each of the C_H_2 domains. The *N*-linked oligosaccharides expressed at this site are the biantennary complex type with microheterogeneities, resulting from the presence or absence of the nonreducing terminal residues, and they have a significant effect on the effector functions of IgG (Fig. 1A) (Burton & Woof 1992; Jefferis *et al.* 1998; Yamaguchi *et al.* 2006, 2007). With regard to the clinical applications of glycoengineered antibodies, the removal of the core fucose residue from the *N*-glycans of IgG-Fc results in dramatic enhancement (>50-fold) of antibody-dependent cellular cytotoxicity (ADCC) through improved IgG binding to Fcγ receptor IIIa (FcγIIIa) (Shields *et al.* 2002; Shinkawa *et al.* 2003; Taniguchi *et al.* 2006; Kubota *et al.* 2009; Yamane-Ohnuki & Satoh 2009). Indeed, a phase I trial of a defucosylated humanized anti-CC chemokine receptor 4 (CCR4) antibody, KW-0761, in relapsed CCR4-positive adult T-cell leukemia–lymphoma or other peripheral T-cell lymphomas revealed that the antibody had antilymphoma activity even at a low dose of 0.01 mg/kg (Niwa *et al.* 2004). Our crystallographic data on uncomplexed IgG1-Fc fragments indicated that the overall structures of the fucosylated and nonfucosylated human IgG1-Fc glycoforms are quite similar except for the hydration mode around Tyr-296 (Matsumiya *et al.* 2007). The crystal structures of the Fc–FcγRIIIb complexes have been reported for fucosylated IgG1-Fc and the soluble form of FcγRIIIb (sFcγRIIIb), which was expressed in *Escherichia coli,* and therefore not glycosylated (Sondermann *et al.* 2000; Radaev *et al.* 2001). The extracellular domains of FcγRIIIa possess five *N*-glycosylation sites at positions 38, 45, 74, 162, and 169 (Ravetch & Perussia 1989). Mutational deglycosylation analyses showed that the *N*-glycan expressed at Asn-162 has negative and positive effects on the binding affinities of the fucosylated and nonfucosylated IgG glycoforms, respectively (Ferrara *et al.* 2006; Shibata-Koyama *et al.* 2009). To gain detailed knowledge of the mechanisms underlying the improvement in ADCC on defucosylation, it is necessary to obtain detailed structural information on the interaction between nonfucosylated IgG and sFcγRIIIa possessing this *N*-glycan.

## Results

### Overall structure of Fc–sFcγRIIIa complex

We solved the crystal structure of the nonfucosylated glycoform of human IgG1-Fc complexed with human sFcγRIIIa glycosylated only at Asn-45 and Asn-162 (Fig. 1B). *N*-glycosylations at these two sites have been reported to be the minimal requirement for the expression of sFcγRIIIa without proteolytic degradation in mammalian cells (Shibata-Koyama *et al.* 2009). The Fc fragment was cleaved from the nonfucosylated IgG1 produced in the *Fut8*^*−/−*^ cell line Ms704 (Matsumiya *et al.* 2007) by papain digestion, whereas the bis-glycosylated sFcγRIIIa was expressed in the CHO/DG44 cell line with the Asn-to-Gln mutation of the remaining glycosylation sites (Shibata-Koyama *et al.* 2009) and treated with sialidase, resulting in homogeneous glycosylation with a biantennary fucosylated complex-type oligosaccharide (Fig. 1A). The crystal structure of nonfucosylated Fc complexed with the bis-glycosylated sFcγRIIIa was determined by a molecular replacement technique with the crystal structure of a human fucosylated IgG1-Fc fragment complexed with nonglycosylated FcγRIIIb (PDB ID code 1E4K) (Sondermann *et al.* 2000) and refined to 2.2-Å resolution (Table 1).

**Table 1:** Data collection and refinement statistics

	Fc–sFcγRIIIa
Data collection statistics	
Source	SP8 BL44XU
Wavelength (Å)	0.900
Resolution (Å)	87.6–2.20 (2.24–2.20)
Space group	*P*4_1_2_1_2
Cell dimensions (Å)	*a*= *b*=77.3, *c*=350.3
Number of observations	673 587
Number of unique reflections	53 300
Redundancy	12.6 (6.9)
Completeness (%)	96.4 (77.4)
Mean *I*/σ (*I* )	20.5 (5.9)
*R*_merge_ (%)	0.089 (0.372)
Refinement statistics	
Resolution (Å)	43.8–2.20
Number of reflections	50 477
Protein atoms	4734
Carbohydrate moieties	338
Water molecules	307
*R*_work_ (%)	22.4
*R*_free_ (%)	26.4
Average *B*-values (Å^2^)	
Protein	42.3
Carbohydrate moieties	61.8
Water	41.4
RMSDs from ideality	
Bond lengths (Å)	0.007
Bond angles (°)	1.03
Ramachandran plot statistics	
Most favored (%)	90.5
Additional allowed (%)	8.9
Generously allowed (%)	0.6
Disallowed (%)	0

Values in parentheses are given for the highest resolution shell. *R*_merge_ = Σ|*I*_j_−<*I*>|/Σ*I*_j_, where *I*_j_ is the intensity of an individual reflection and <*I*> is the average intensity of the reflection. *R*_work_ = Σ||*F*_o_|−|*F*_c_||/Σ|*F*_o_|, where *F*_c_ is the calculated structure factor. *R*_free_ is as for *R*_work_, but calculated for a randomly selected 5.0% of reflections not included in the refinement. sFcγRIIIa, soluble form of Fcγ receptor IIIa.

The overall structure of the present Fc–sFcγRIIIa complex is similar to previously reported crystal structures of the complexes formed between glycosylated Fc and nonglycosylated sFcγR IIIb (1E4K, 1T83, and 1T89) (Radaev *et al.* 2001; Sondermann *et al.* 2000; Fig. S1 in Supporting Information): The hinge region and both C_H_2 domains are involved in the interaction with the D2 domain of sFcγRIIIa, and Fc undergoes a conformational change to adopt a slightly more open conformation. The electron density of the *N*-glycans linked to Fc was well defined and enabled us to locate eight sugar residues (GlcNAc-1, GlcNAc-2, Man-3, Man-4, Man-4′, GlcNAc-5, GlcNAc-5′, and Gal-6′) on Fc chains A and B (Fig. 1). In the case of sFcγRIIIa, electron densities of three (GlcNAc-1, GlcNAc-2, and Man-3) and eight (Fuc, GlcNAc-1, GlcNAc-2, Man-3, Man-4, Man-4′, GlcNAc-5, and GlcNAc-5′) sugar residues were observed in the *N*-glycans displayed at Asn-45 and Asn-162, respectively (Fig. 1).

### Fc–sFcγRIIIa interface

The present crystal structure exhibits a contact surface area of 1218 Å^2^, which is larger than those observed in the previously reported crystal structures of the complexes formed between fucosylated Fc and nonglycosylated sFcγRIIIb (876 Å^2^ in 1E4K, 887 Å^2^ in 1T83, and 800 Å^2^ in 1T89). In the present crystal structure, the surface areas occupied by the carbohydrate moieties and the protein portion of the Fc are 261 and 957 Å^2^, respectively. The sFcγRIIIa-binding sites consist of the lower hinge, B strand, loop B/C, loop D/E, and loop F/G of Fc chain A as well as the lower hinge, B strand, and loop F/G of Fc chain B (Table 2).

**Table 2:** Interactions between immunoglobulin G (IgG)-Fc and soluble form of Fcγ receptor IIIa (sFcγRIIIa)

sFcγRIIIa	Fc chain A	Fc chain B
E21		P329
I88		P329, A330
G89		P329
W90		L235, G236, G237, A327, L328, P329
W113		L235, K326, A327, L328, P329,
K114		
T116		L235
A117		L235
H119	L235, G236	
K120	G236, G237, P238, S239, D265, GN1	
T122	N297, GN1	
G127	Y296	
K128	E294, Q295, Y296, N297, S298	
G129	Y296, N297, S298	
R130	S298	
K131	E269, H268, S298	
Y132	G237, P238, D265, V266, S267, E269, S298, T299, GN1	
H134	G236, G237, P238, D265, V266, S267, N325, A327	
H135	L235, G236	
R155	N297, GN1	
L157	L235	
V158	L235, G236	
G159	L235, G236	
K161	G236, G237, P238, S239, I332	
GN1	GN1	
GN2	GN1	
M3	Y296	
M4	M4′	
M4′	Y296	
GN5	R301, GN2, M4′	
GN5′	Y296	
Fuc		GN5

The underlined residues are involved in hydrogen-bonding interactions.

**Figure 2:**
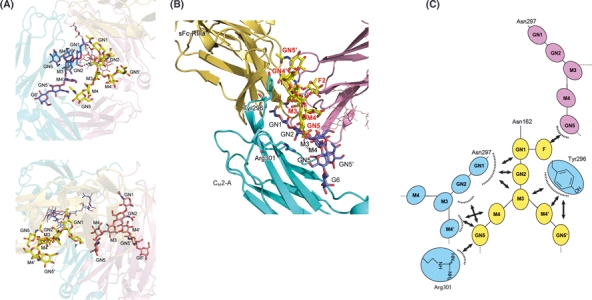
Binding of nonfucosylated Fc to soluble form of Fcγ receptor IIIa (sFcγRIIIa) is mediated by carbohydrate–protein and carbohydrate–carbohydrate interactions. (A) Close-up view of the interface between sFcγRIIIa-Asn162 *N*-glycan and the Fc *N*-glycans [in chain A (upper panel) and chain B (lower panel)]. Hydrogen bonds are represented by dotted lines. (B) Close-up view of the interface between the sFcγRIIIa-Asn162 *N*-glycan (yellow) and Fc chain A (cyan) (C) Schematic representation of the carbohydrate–carbohydrate interactions.

On the receptor side, the Fc contact surface is composed of the loops located in the D2 domain. Although the D1 domain of sFcγRIIIa makes little direct contacts with Fc, the carbohydrate chain linked to Asn-45 is oriented toward the C_H_2 domain of Fc chain B (Table 2). Interestingly, the *N*-glycan at Asn-162 interacts with the Fc *N*-glycans, particularly those of chain A, through hydrogen bonds and van der Waals contacts (Fig. 2). The carbohydrate–carbohydrate interaction occupies approximately 12% (145 Å^2^) of the total interface area.

**Figure 3:**
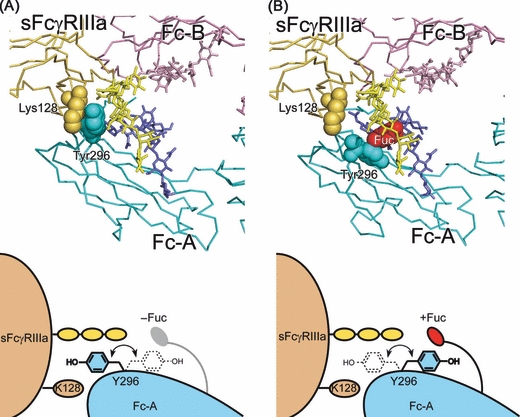
Alternative modes of Fc–soluble form of Fcγ receptor IIIa (sFcγRIIIa) interaction with different orientations of the Tyr-296 aromatic ring depending on Fc fucosylation. (A) The present crystal structure, in which the aromatic ring of Tyr-296 of nonfucosylated Fc (chain A) is flipped out and sandwiched between Man-4 of the Asn-162 glycan and Lys-128 of sFcγRIIIa. (B) 3D model of sFcγRIIIa bound to fucosylated Fc, in which the tyrosine ring does not make contact with the receptor, but makes intramolecular contact with the core fucose residue. The model is based on the crystal structure of the complex between fucosylated Fc and nonglycosylated sFcγRIIIb (1E4K) with substitution of the receptor molecule by bis-glycosylated sFcγRIIIb in the present crystal structure by superposing its D2 domains. In this model, the core fucose residue was attached to GlcNAc-1 by the α1–6 glycosidic linkage in the *N*-glycans in Fc chain A by superimposing the crystal structure of the fucosylated IgG1-Fc (3AVE). Chains A and B of Fc are cyan and pink, respectively, and sFcγRIIIa is yellow.

The present crystal structure also shows that the aromatic ring of Tyr-296 of Fc chain A is flipped out and forms a hydrogen bond and van der Waals contacts with Man-4 of the Asn-162 *N*-glycan as well as Lys-128 of sFcγRIIIa (Fig. 3), thereby stabilizing the complex. Surface plasmon resonance (SPR) data consistently indicated that alanine substitution of Tyr-296 resulted in impaired affinity for sFcγRIIIa in both fucosylated and nonfucosylated IgG1 glycoforms (Fig. S2 in Supporting Information). In the previously reported crystal structures of fucosylated Fc and nonglycosylated sFcγRIIIb (i.e., 1E4K, 1T83, and 1T89), the D/E loop of Fc chain A, which contains this tyrosine residue, exhibits two alternate conformations. In one crystal structure (1T83), which bears the closest resemblance in overall structure to the present structure, the Tyr-296 aromatic ring is also flipped out, interacting with Lys-128 of sFcγRIIIb. In the remaining crystal structures (1E4K and 1T89), this tyrosine ring makes intramolecular contacts with the core fucose residue and turns away from sFcγRIII. These data suggest that Fc and sFcγRIII have two binding modes with high and low affinities, depending on the orientation of the aromatic ring of Tyr-296 (Fig. 3).

### Involvement of carbohydrate moieties in Fc–sFcγRIIIa interactions

**Figure 4:**
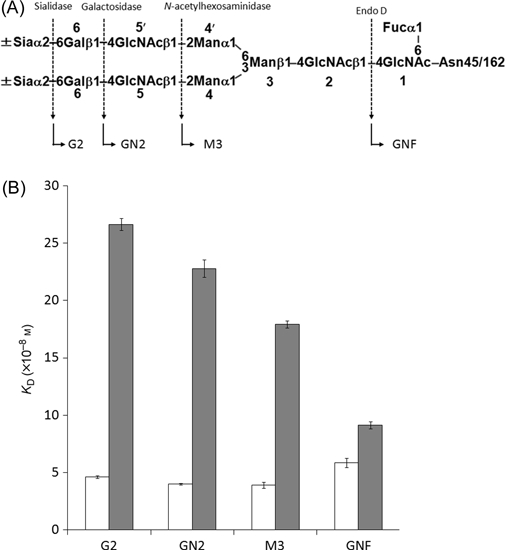
Glycoform-dependent interactions between human immunoglobulin G1 (IgG1) and soluble form of Fcγ receptor IIIa (sFcγRIIIa). (A) Carbohydrate sequence of sFcγRIIIa used in the present study. Dotted arrows indicate cleavage sites of glycosidases. The resulting glycoforms are indicated by arrows. (B) White and gray bars represent *K*_D_ values of the binding of sFcγRIIIa glycoforms to nonfucosylated and fucosylated IgG glycoproteins, respectively. These values were calculated based on SPR data.

**Figure 5:**
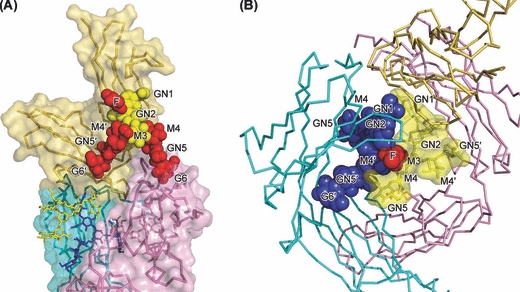
Potential steric hindrance effects in interactions between (A) C_H_2 domain of Fc and soluble form of Fcγ receptor IIIa (sFcγRIIIa)-Asn-45 *N*-glycan and (B) fucosylated *N*-glycan of Fc chain A and sFcγRIIIa-Asn-162 *N*-glycan. The sugar residues of the Asn-45 *N*-glycan gave no interpretable electron density, and the core fucose residues of the Fc *N*-glycan were modeled and displayed in red. The modeling was based on the conformations of the *N*-glycan of Fc chain A (for the lactosamine branches in A) and the Asn-162 *N*-glycan (for the fucose residues in A and B).

To assess the importance of the observed carbohydrate–carbohydrate interactions, we investigated the possible effects of enzymatic trimming of the *N*-glycans of sFcγRIIIa on its binding to fucosylated and nonfucosylated IgG glycoproteins by SPR measurements (Fig. 4). The Fc binding affinity slightly increased on the removal of the outer lactosamine branches of the sFcγRIIIa *N*-glycans, although GlcNAc-5 of the Asn-162 *N*-glycan positively interacts with Man-4′ and Arg-301 of Fc chain A, at least in the case of its nonfucosylated glycoform (Fig. 2B,C). This affinity enhancement can be ascribed to the removal of the outer carbohydrate branches of the *N*-glycan at Asn-45, which are in close spatial proximity to the C_H_2 domain of Fc chain B and may cause steric hindrance, although their electron densities are not interpretable (Fig. 5A). Mutational deglycosylation at Asn-45 was reported to enhance the affinity for Fc (Shibata-Koyama *et al.* 2009).

The removal of GlcNAc-2 and the trimannosyl parts of the sFcγRIIIa *N*-glycans (i.e., Man-3, Man-4, and Man-4′) have more pronounced positive and negative effects on the interactions with fucosylated and nonfucosylated Fc glycoforms, respectively (Fig. 4). These data are consistent with previously reported mutational deglycosylation data (Ferrara *et al.* 2006; Shibata-Koyama *et al.* 2009), as well as the present crystal structure in which these sugar residues show productive contacts with Tyr-296 and the nonfucosylated GlcNAc-1 residue of Fc chain A. Furthermore, our structural model indicates that fucosylation of GlcNAc-1 in chain A causes steric hindrance with GlcNAc-2 and Man-3 of the sFcγRIIIa-Asn-162 *N*-glycan (Fig. 5B), which results in impaired interaction between these two glycoproteins. This explains why affinity enhancement on the extensive trimming of the sFcγRIIIa *N*-glycans was selectively observed in the fucosylated glycoform of Fc.

## Discussion

Although it is widely recognized that glycosylation of immune receptors can influence their affinities for the cognate ligands (Standley & Baudry 2000), not much is known about the specific roles of the individual glycans from a structural aspect. The present crystallographic data demonstrated that one of the two *N*-glycans (Asn162 *N*-glycan) of sFcγRIIIa mediates the interaction with nonfucosylated Fc, thereby stabilizing the complex. To the best of our knowledge, this is the first atomic description of the carbohydrate–carbohydrate interactions that mediate the formation of complexes between glycoproteins. Fucosylation of the Fc *N*-glycans inhibits these positive interactions, because of steric hindrance, thereby impairing IgG binding to FcγRIIIa and the consequent ADCC activity. On the other hand, the *N*-glycan displayed at Asn-45 of sFcγRIII negatively affects its binding to Fc (Shibata-Koyama *et al.* 2009), most probably because of steric clash with the C_H_2 domain (Fig. 5A).

Moreover, the present crystal structure, in comparison with those previously reported for the low-affinity complexes, indicates that Fc and sFcγRIII have two binding modes depending on the orientation of the aromatic ring of Tyr-296 of Fc chain A (Fig. 3). Our previous NMR data demonstrated that Tyr-296 of the nonfucosylated Fc glycoform exhibits conformational multiplicity in its uncomplexed state (Matsumiya *et al.* 2007), suggesting that conformational selection is governed by the presence or absence of the fucose residue of the Fc *N*-glycan. Fucose depletion increases the incidence of the active conformation of Tyr-296, and thereby accelerates the formation of the high-affinity complex. This interpretation is supported by our previous data indicating that an increase in affinity by defucosylation was primarily ascribed to an enhanced association rate (Okazaki *et al.* 2004). Consistently, our SPR data show that sFcγRIIIa has higher affinity for the nonfucosylated Fc than for the fucosylated Fc, even after extensive trimming of its *N*-glycans (Fig. 4).

These data are consistent with previously reported thermodynamic data indicating that affinity enhancement on defucosylation is characterized by favorable Δ*H,* but opposed by unfavorable Δ*S* (Okazaki *et al.* 2004). The favorable Δ*H* can be ascribed, at least partially, to the productive contacts caused by the flipping out of the Tyr-296 ring and accommodation of the sFcγRIIIa-Asn-162 *N*-glycan, both of which result in conformational entropy loss.

The present crystallographic data provide a structural basis for the improvement in ADCC on defucosylation of IgG-Fc through the enhancement of its affinity for FcγRIIIa, thus offering new clues for designing and engineering antibody medicines with improved efficacy. From a more general viewpoint, this study shows that the oligosaccharides displayed on proteins can modulate complex formation, positively and negatively, not only through intermolecular carbohydrate–protein and carbohydrate–carbohydrate interactions but also by influencing protein dynamics coupled with the selection of protein–protein interaction modes.

## Experimental procedures

### Preparation of IgG-Fc

The CHO/DG44 cell line (Urlaub *et al.* 1986) was kindly provided by Dr Lawrence Chasin (Columbia University, NY). The fucosylated and nonfucosylated forms (designated KM3060 and KM3416, respectively) of an anti-CCR4 chimeric antibody with human IgG1/κ constant regions along with their Y296A mutants, constructed using the QuikChange® Multi Site-Directed Mutagenesis kit (Stratagene), were expressed by the CHO/DG44 cell line and the *FUT8*^*−/−*^ cell line Ms704, respectively, as previously described (Matsumiya *et al.* 2007). The nonfucosylated and fucosylated IgG1 glycoproteins were expressed and purified as previously described (Yamaguchi *et al.* 2006; Matsumiya *et al.* 2007). The Fc fragments were prepared by papain digestion as previously described (Yamaguchi *et al.* 2006), and the purity of the isolated Fc fragment was examined by SDS-PAGE.

### Preparation of sFcγRIIIa

The human sFcγRIIIa mutant with two *N*-glycosylation sites at Asn-45 and Asn-162 was expressed and purified as previously described (Shibata-Koyama *et al.* 2009). In brief, the extracellular region of human FcγRIIIa with an N-terminal hexahistidine tag and glutamine substitutions at Asn-38, Asn-74, and Asn-169 was expressed by the CHO/DG44 cell line as a recombinant protein modified exclusively with sialylated biantennary *N*-glycans (Shibata-Koyama *et al.* 2009). A series of sFcγRIIIa glycoforms were prepared according to the protocols described below.

1. Sialidase treatment: sFcγRIIIa was dissolved in 50 mm sodium acetate buffer, pH 5.5, and incubated at 37 °C for 12 h in the presence of 0.01 units/mL α-sialidase (*Arthrobacter ureafaciens*; Nacalai Tesque).
2. Galactosidase treatment: Desialylated sFcγRIIIa was dissolved in 50 mm sodium acetate buffer, pH 5.5, and incubated at 37 °C for 12 h in the presence of 0.006 units/mL of β-galactosidase (*Streptococcus pneumoniae*, recombinant protein from *E. coli*; Calbiochem).
3. *N*-Acetylhexosaminidase treatment: Degalactosylated sFcγRIIIa (2 mg/mL) was dissolved in 50 mm sodium acetate buffer, pH 5.5, and incubated at 37 °C for 12 h in the presence of 0.1 units/mL of *N*-acetylhexosaminidase (*S. pneumoniae*, recombinant protein from *E. coli*; Calbiochem).
4. Endo D treatment: Galactosidase- and *N*-acetylhexosaminidase-treated sFcγRIIIa (2 mg/mL) were dissolved in 50 mm sodium acetate buffer, pH 5.5, and incubated at 37 °C for 12 h in the presence of 0.02 units/mL of Endo D (*Diplococcus pneumoniae*; Seikagaku Kogyo Co.).

In each case, the reaction mixture was neutralized with 1.5 m Tris–HCl (pH 8.0) and then subjected to Ni-NTA chromatography to purify sFcγRIIIa. sFcγRIIIa was incubated under the same conditions in the absence of the corresponding enzyme(s) to prepare mock-treated controls.

### Glycosylation profiling

*N*-glycosylation profiling of Fc and sFcγRIIIa was carried out by the HPLC mapping method on the basis of elution profiles of pyridylamino derivatives of their *N*-linked oligosaccharides on Shim-pack HRC-octadecyl silica columns (Shimadzu), as previously described (Takahashi *et al.* 2002; Yamaguchi *et al.* 2006).

### Crystallization, data collection, and structure determination

The nonfucosylated Fc fragment and desialylated sFcγRIIIa mutant were mixed at a molar ratio of 1 : 1 and then applied to a gel filtration column (Superose 12; GE Healthcare) equilibrated with 20 mm Tris–HCl buffer, pH 7.5, containing 100 mm NaCl. Fractions containing the Fc–sFcγRIIIa complex were concentrated to a total protein concentration of 20 mg/mL and used for crystallization.

Crystals were grown by the sitting-drop vapor-diffusion method at 20 °C by mixing the Fc–sFcγRIIIa complex with a reservoir [0.1 m MES, pH 6.5, 12% (w/v) PEG 20000]. Crystals were soaked in a cryoprotectant solution [0.1 m MES, pH 6.5, 20% (w/v) PEG 20000, 15% (v/v) glycerol] and flash-frozen. Diffraction data were collected at 100 K using a wavelength of 0.9 Å on a beamline BL44XU (SPring-8). Data processing and reduction were carried out using the HKL-2000 software package (Otwinowski & Minor 1997). The crystals belong to the space group *P*4_1_2_1_2, with cell dimensions *a*= *b*=77.3 Å, *c*=350.3 Å at 2.2-Å resolution. The value of the Matthews coefficient is 3.34 Å^3^/Da for one molecule, corresponding to a solvent content of 63.2%. Data collection, phasing, and refinement statistics are summarized in Table 1. The structure of the complex between nonfucosylated Fc and bis-glycosylated sFcγRIIIa was determined by molecular replacement using the structures of the complex between fucosylated Fc and nonglycosylated sFcγRIIIa (Sondermann *et al.* 2000) (PDB ID code 1E4K) using MOLREP(CCP4, 1994; Vagin & Teplyakov 1997). The model was furthermore built using the program COOT (Emsley & Cowtan 2004) and then improved by several cycles of manual rebuilding and refinement using the program REFMAC5 (Murshudov *et al.* 1997). The final model contains the Fc residues 229–443 (chain A) and 229–444 (chain B), and the sFcγRIIIa residues 5–30 and 41–174. Phasing and refinement statistics are summarized in Table 1. There are no residues in disallowed regions of the Ramachandran plot. Buried surface area and surface complementarity were calculated using AREAIMOL (Lee & Richards 1971). SFCHECK and PROCHECK (CCP4, 1994) were used for structure validation. Molecular graphics were prepared using PyMOL (DeLano 2002). Atomic coordinates and structure factors have been deposited in the PDB under accession code 3AY4.

### Surface plasmon resonance measurements

Interactions of the sFcγRIIIa glycoforms with fucosylated and nonfucosylated IgG glycoproteins were analyzed by SPR using the T100 biosensor system (GE Healthcare). Mouse anti-tetra-His IgG antibody (Qiagen) was immobilized on CM5 biosensor chips by an amine coupling method according to the manufacturer's instructions. The individual glycoforms of the hexa-His-tagged sFcγRIIIa glycoproteins were captured by the immobilized anti-tetra-His antibodies at a flow rate of 5 μL/min using HBS-EP+ buffer (10 mm HEPES, 150 mm NaCl, 3 mm EDTA, and 0.05% v/v surfactant P20, pH 7.4) at 25 °C. Assays were performed using nonfucosylated and fucosylated IgG glycoproteins at seven concentrations (ranging from 4 to 267 nm) in a mobile phase at a flow rate of 30 μL/min using the HBS-EP+ buffer at 25 °C. The dissociation constant (*K*_D_) was calculated by steady-state affinity analysis using Biacore T100 evaluation software version 2.0.1 (GE Healthcare). To repeat experiments, sFcγRIIIa and human IgG1 were removed from the sensor tips by injecting 10 mm glycine–HCl, pH 1.5, at a flow rate of 60 μL/min for 1 min. *K*_D_ values are the mean ± SD of three independent experiments.

## Supporting Information / Supplementary material

The following Supporting Information can be found in the online version of the article:

**Figure S1** Superposition of the present crystal structure of the complex between IgG–Fc (cyan and pink) and sFcγRIIIa (yellow) and the structures previously reported for the IgG–Fc–sFcγRIIIb complexes (gray).

**Figure S2** Contribution of Tyr–296 of human IgG1 to its interactions with sFcγRIIIa.
