# Supplementary material for: Structural basis for improved efficacy of therapeutic antibodies on defucosylation of their Fc glycans
Source: Genes Cells. 2011 Nov;16(11):1071–80. doi: 10.1111/j.1365-2443.2011.01552.x (PMC3258418; doi:10.1111/j.1365-2443.2011.01552.x)
Supplement: Supplementary file 1 [file gtc0016-1071-SD1.doc]

**Supplementary material**

**Figure S1.**

**Figure S1. Superposition of the present crystal structure of the complex between IgG-Fc (cyan and pink) and sFcγRIIIa (yellow) and the structures previously reported for the IgG-Fc-sFcγRIIIb complexes (grey).** (A) 1E4K, (B) 1T83, and (C) 1T89. The D/E loop of Fc chain A of the present crystal structure is red and that of the previously reported structures is orange.

**Figure S2.**

**Figure S2. Contribution of Tyr-296 of human IgG1 to its interactions with sFcγRIIIa.** The white and black bars represent the *K*_D_ values of the bindings of sFcγRIIIa to wild-type and Y296A mutants of human IgG1 (in fucosylated or non-fucosylated form). These values were calculated based on SPR data.
